# Asymptomatic Cryptococcal Antigenemia: A Multi-clinic Cross-Sectional Study on the Prevalence, Associated Factors and Predictors of Three-Month Outcomes Among Antiretroviral-Naïve and Antiretroviral-Experienced HIV Patients in Dar es Salaam, Tanzania

**DOI:** 10.7759/cureus.98852

**Published:** 2025-12-09

**Authors:** Mandela Makakala, Mukasa Mohammed, Mabula M Mabelele, Kamran Hameed, Philip B Adebayo

**Affiliations:** 1 Internal Medicine, Aga Khan University, Dar es Salaam, TZA; 2 Neurology, Aga Khan University, Dar es Salaam, TZA

**Keywords:** aids, antiretroviral-experienced, antiretroviral-naive, antiretroviral therapy, asymptomatic cryptococcal antigenemia, cd4 count, hiv, viral load

## Abstract

Background: Cryptococcal meningitis remains a leading cause of HIV/AIDS-related mortality, predominantly in Sub-Saharan Africa. Current screening and prevention efforts primarily target antiretroviral therapy (ART)-naïve patients, whereas strategies for ART-experienced individuals are less well defined.

Objectives: This study primarily aimed at determining the prevalence of asymptomatic cryptococcal antigenemia (ACA) among ART-naïve and ART-experienced patients, along with associated factors and predictors of three-month outcomes among HIV patients in three HIV clinics in Dar es Salaam, Tanzania. Secondarily, factors associated with serum cryptococcal antigen positivity in the overall study population were also assessed.

Methods: This cross-sectional clinic-based study recruited 273 HIV-positive participants (ART-naïve and ART-experienced), attending three HIV care clinics in Dar es Salaam over a period of three months. A convenient sampling technique was employed, and all eligible participants were recruited until the desired sample size was reached. The study included adults (≥18 years) with recent CD4 cell counts of ≤200 cells/μL and ART-experienced patients (on ART treatment for more than six months) who had a viral load ≥1000 copies/mL. Individuals with symptoms of meningitis and recent fluconazole use were excluded. Screening for serum cryptococcal antigen (CrAg) was performed in all participants. The predetermined three-month outcomes, including development of meningitis, hospitalization, and mortality, were assessed.

Results: The overall prevalence of ACA was 4.8% (13/273; 95% CI: 2.6-8.0). Among ART-experienced patients, the prevalence was 2.5% (4/162; 95% CI: 0.7-6.2), compared to 8.1% (9/111; 95% CI: 3.8-14.8) in the ART-naïve patients (p < 0.05). At three months, the likelihood of death, development of cryptococcal meningitis, or hospitalization was significantly higher among CrAg-positive patients (69%) compared to CrAg-negative patients (p < 0.05). Overall, a history of headache within the preceding two weeks and hospitalization in the past 12 months were significantly associated with CrAg positivity.

Conclusion: The overall prevalence of ACA across the three HIV clinics in Dar es Salaam was 4.8%, with lower prevalence observed among ART-experienced patients compared to ART-naïve individuals. CrAg positivity was a strong predictor of adverse outcomes at three months in both populations. These findings underscore the need for more robust, risk-based guidelines on screening and surveillance even among ART-experienced patients.

## Introduction

*Cryptococcus neoformans* is a leading cause of meningitis among adults in Sub-Saharan Africa [1,2] and is responsible for approximately 20% of human immunodeficiency virus (HIV)-related mortality globally, with the majority of these deaths occurring in Sub-Saharan Africa [3]. Despite its substantial burden, it remains one of the neglected tropical diseases [4].

The prevention and management of cryptococcal meningitis face numerous challenges, especially in developing countries, due to limited diagnostic capacity, scarcity of essential medication, and shortage of physicians [5-7]. Comprehensive HIV care is also a cornerstone in the prevention of cryptococcal meningitis. With global improvements in HIV care and broader antiretroviral therapy (ART) coverage, a corresponding decline in cryptococcal burden would be expected [8]. However, despite the advances in treatment and expanding ART access, the prevalence and mortality rate of cryptococcal meningitis have remained significant [8,9]. This has shifted the focus toward prevention in HIV patients, specifically those with low CD4 (<100 cells/µL) [10], by screening and preemptive treatment of asymptomatic cryptococcal antigenemia (ACA) [11]. ACA, which is the presence of cryptococcal species in the blood without having symptoms of meningitis (fever, headache, stiff neck, and altered mental status), is a subclinical disease that may progress to cryptococcal meningitis within three to six weeks if left untreated [12]. In a study conducted in rural Uganda, Tanzania’s western neighbor, ACA was found to independently predict death during the first three months of ART among individuals with advanced HIV disease [12].

The WHO guideline, a major reference in HIV care in Sub-Saharan Africa, currently recommends routine screening for cryptococcal antigenemia in ART-naïve HIV patients with CD4 cell counts ≤ 100 cells/µL on initiation or reinitiation of treatment, but only conditionally does so for those with CD4 between 100 and 200 cells/µL. Even with these clear recommendations, the incorporation of routine cryptococcal antigen (CrAg) screening into HIV care has remained a global challenge [4]. On a positive note, however, this guidance has led to greater emphasis on screening among ART-naïve patients, as reflected in most studies conducted both regionally and globally, for example, in Namibia [13], west Africa (Benin and Nigeria) [14], Indonesia [15], Uganda [16], UK [17], and Iran [18]. In contrast, relatively few studies have focused on ART-experienced cohorts, most of which were conducted in Ethiopia [19-21]. Similarly, in Tanzania, most research on cryptococcal infection has focused on symptomatic disease [7,22] or ART-naïve patients [23].

Although cryptococcal antigenemia has been reported to be nearly 100% sensitive in predicting the development of cryptococcal meningitis [24], regardless of ART experience, there are still no specific recommendations or guidelines for screening ART-experienced patients. This gap persists despite emerging evidence of substantial disease burden in this group, as demonstrated by Alemu et al. [19], who reported that 84% of patients with cryptococcal antigenemia were already receiving ART.

With regard to the risk factors associated with cryptococcal antigenemia, a low CD4 count is well established; however, findings on other potential factors have been inconsistent. For instance, in one study, a BMI less than 18 kg/m^2^ (p = 0.0007) was the only significant factor associated with cryptococcal antigenemia, but anemia and high viral load were not [12]. In other studies, however, BMI of less than 15.4 kg/m^2^, being male, rural residence, and being hospitalized were all associated with cryptococcal antigenemia [16,20]. One study in Nigeria, which assessed the association of ART regimen with CrAg positivity, reported that no factors were significantly associated with cryptococcal antigenemia [25].

The primary objective of this study, therefore, was to determine the prevalence of ACA and development of meningitis among ART-naive patients with CD4 counts ≤ 200 cells/µL and ART-experienced patients with HIV viral load of ≥ 1000 copies/mL, recruited from three HIV clinics in Dar es Salaam, Tanzania. Secondarily, it aimed to determine the factors associated with the ACA and its short-term outcomes (development of meningitis, deaths, and number of hospitalizations). Predictors of CrAg positivity in the study cohort were also investigated.

## Materials and methods

Study setting and design

At the time of the study, 1.4 million people were living with HIV in Tanzania, with a prevalence of 4.7% and there were 55,000 new infections per year [26]. The study was conducted in Tanzania’s commercial capital city, Dar es Salaam, which is the most populous city in Tanzania (according to the 2012 national census), with a population of approximately 4.3 million people and an HIV prevalence of about 4.7% in the region [27].

This was a cross-sectional, multi-center, clinic-based study. The study included HIV-positive adults from three HIV care and treatment clinics (CTCs) located in their respective hospitals: Mwananyamala Hospital, Mnazi Mmoja Hospital (both public), and Aga Khan Hospital (private). These clinics are part of the national HIV care network overseen by the Ministry of Health of Tanzania. The study assessed the prevalence of ACA among eligible patients and followed CrAg-positive individuals for 12 weeks to evaluate short-term outcomes (development of meningitis symptoms, hospitalization, or death).

Study population and eligibility criteria

The study population included HIV-positive adults (aged ≥18 years), both ART-naïve and ART-experienced, attending the selected CTCs from September 2018 to February 2019. Eligibility criteria included a documented recent CD4 count ≤ 200 cells/μL within the past three months, and for ART-experienced individuals, they must have been on ART treatment for more than six months, with a recent viral load ≥1000 copies/mL. Exclusion criteria included current or recent symptoms of cryptococcal meningitis (defined as having two or more of the following: fever, headache, neck stiffness, and altered mental status), use of fluconazole within the preceding two weeks, a history of cryptococcal infection within the past year, or an inability to complete 12-week follow-up.

In this study, an ART-experienced patient was defined as an HIV-positive individual on ART for more than six months, while an ART-naïve patient was defined as one who had never received ART or had been on ART for six months or less. ACA was defined as a positive serum CrAg test in an individual without clinical symptoms of cryptococcal meningitis (not more than one of the following: headache, fever, neck stiffness, altered mentation, convulsions, or photophobia/blurred vision). Recent CD4 count and viral load referred to measurements obtained within the preceding three months.

Sample size and sampling strategy

The prevalence of cryptococcal antigenemia from a prior clinic-based study in Dar es Salaam was found to be 5%. However, this study was conducted in ART-naive patients only, and the prevalence was comparable to findings from similar studies conducted in Indonesia [15] and South Africa [24], which reported the prevalences of 7.1% and 7%, respectively. The study [21] conducted in Ethiopia, which included both ART-naïve and ART-experienced patients, reported the prevalence of cryptococcal antigenemia to be 10.2%.

The sample size was calculated using the formula for estimating a population proportion, with a prevalence estimate of 10.2% based on previous studies in ART-experienced populations. Using a 95% confidence interval and a 4.2% margin of error, the minimum required sample was 220 participants. To account for potential attrition (estimated at 20%), the final target sample size was set at 265 participants. The formula for sample size calculation was, n = (Z2 x P (1 - P))/e2, where Z = value from the standard normal distribution corresponding to the desired confidence level (Z = 1.96 for 95% CI).

A convenience sampling method was employed. Eligible patients attending the CTCs during the study period were consecutively enrolled until the desired sample size was reached. All CrAg-positive participants were followed up for 12 weeks. For outcome comparison, a random sample of CrAg-negative participants was selected at a 2:1 ratio using simple random sampling, whereby for every one CrAg-positive patient, two CrAg-negative patients were followed up for 12 weeks since the date of enrolment and assessed for death, hospitalization, and development of symptoms of meningitis.

Data collection and variables

Data were collected through structured interviews and review of patient medical records. Variables included demographic data (age and sex), clinical characteristics (weight, height, BMI, duration of HIV infection, ART duration, CD4 count, viral load, and ART regimen), and history of comorbidities (e.g., diabetes, hypertension, malignancy, and renal failure). The presence of opportunistic infections or AIDS-defining illnesses was also recorded.

Cryptococcal antigenemia was assessed using a lateral flow assay (LFA) kit from IMMY Diagnostics (Norman, OK). The WHO guideline had recommended the LFA technique in the screening of cryptococcal antigenemia due to its high sensitivity (95-100%) and specificity (100%). A 2 mL venous blood sample was collected from each participant and tested according to the manufacturer's instructions. The sample was collected on the day of the patient visit after the interview, and the CrAg testing was conducted immediately after sample collection. After recording the results of CrAg, the samples were discarded in the biohazard boxes as per facility protocol and later disposed of by incineration according to the specific facility protocol. No sample was stored for future testing in this study.

**Figure 1:**
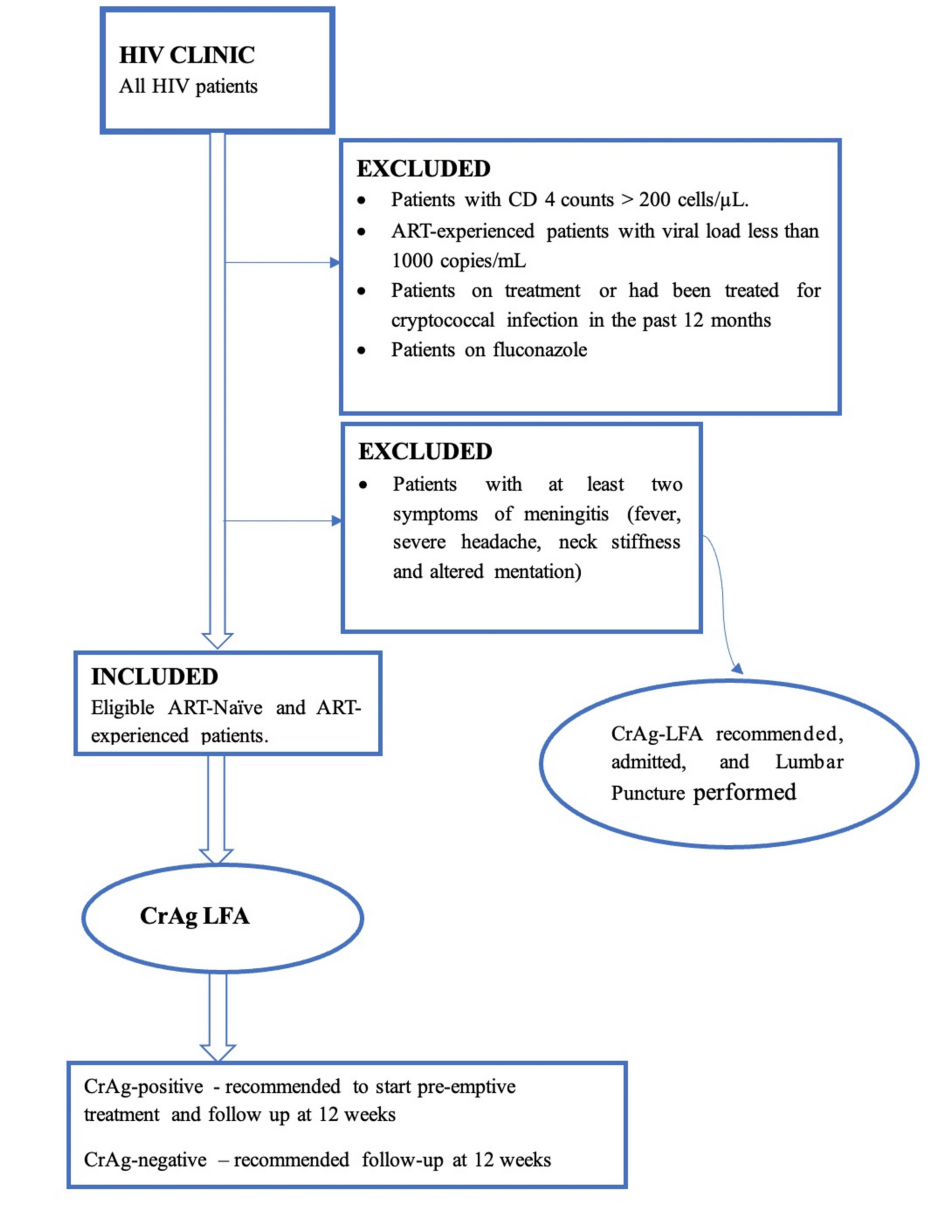
Flow diagram of the study protocol. ART: antiretroviral therapy; CrAg: cryptococcal antigen; LFA: lateral flow assay.

Follow-up assessments for CrAg-positive individuals and the selected CrAg-negative controls were conducted at 12 weeks post-enrollment through phone interviews with patients or their next of kin. During data collection, patients provided their mobile numbers and mobile numbers of the next of kin, and they were told to expect a phone call from the investigator after three months. At three months follow-up, the investigator called the study participants, and, if unavailable, the next of kin was called to provide specific information about the development of symptoms, whether or not they were admitted in the past three months, and the death of the participant. Outcomes included the development of meningitis symptoms, hospitalization, and death. Figure 1 illustrates the recruitment protocol.

Data management and statistical analysis

Data were entered into Epi Info version 7.2.2.16 (Centers for Disease Control and Prevention, Atlanta, GA) and analyzed using the same software. Descriptive statistics were used to summarize baseline characteristics. Categorical variables were presented as frequencies and percentages, and continuous variables as means with standard deviations. The prevalence of ACA was reported as the proportion of CrAg-positive cases in the total sample.

Associations between ACA and independent variables (Table 1) were assessed using chi-square or Fisher’s exact test for categorical variables, and Student’s t-test for continuous variables. Odds ratios (ORs) with 95% confidence intervals (CIs) were calculated. Variables with p-values < 0.05 in the univariate analysis were included in a multivariate logistic regression model to identify independent predictors of ACA. Comparisons of 12-week outcomes between CrAg-positive and CrAg-negative groups were made using chi-square tests.

**Table 1:** Dependent and independent variables. ART: antiretroviral therapy; CrAg: cryptococcal antigen; LFA: lateral flow assay.

Dependent variables	Independent variables
CrAg LFA status	CD4 cell count
	Viral load
	BMI
	History of hospital admission in the past 12 months
	Duration of HIV treatment
	Co-existing HIV-associated illness
	ART adherence
	Cryptococcal meningitis (development of at least 2 symptoms of meningitis)
	Death
	Hospitalization

## Results

Population characteristics

**Figure 2:**
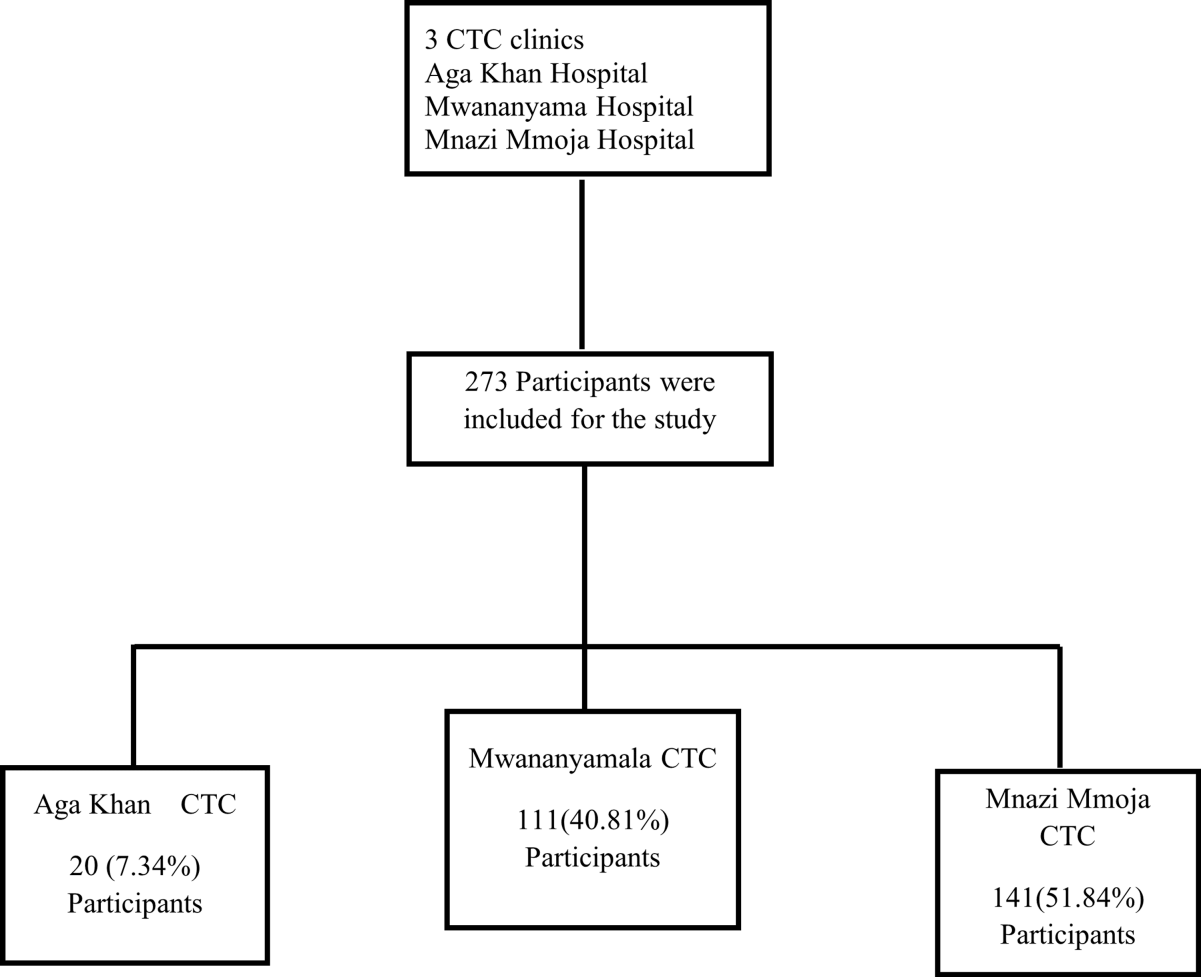
Summary of the sources of the study participants. CTC: care and treatment clinic.

From September 2018 to February 2019, a total of 273 participants were enrolled in the study from three different CTC clinics: Mnazi Mmoja, Mwananyamala, and Aga Khan Hospital. Figure 2 shows a summary of study participants who met the inclusion criteria and consented to participate in the study, and Table 2illustrates the baseline characteristics of the study participants.

**Table 2:** Baseline characteristics of the study participants in the three HIV clinics (N = 273). ART: antiretroviral therapy.

Characteristics		ART-naïve (n = 111, 40.7%)	ART-experienced (n = 162, 59.3%)	p-value
		n (%)	n (%)	
Age (years), mean (SD) = 40.4 (9.9)		40.6 (10.1)	40.4 (9.9)	0.8709
Sex (%)	Male = 97 (35.5)	44 (39.6)	53 (32.7)	0.2958
	Female = 176 (64.5)	67 (60.4)	109 (67.3)	0.2958
BMI, mean (SD) = 23 (4.9)		22.7 (4.6)	23.2 (5.1)	0.4086
CD4 count, mean (SD)		98 (63)		
Viral load (copies/ml), mean (SD)			179033 (761614)	
Marital status				
Married		123 (45.2)		
Single		92 (33.8)		
Divorced/separated		32 (11.8)		
Widowed		25 (9.2)		
Education level		n (%)		
None		15 (5.5)		
Primary education		147 (54.0)		
Secondary education		76 (27.9)		
University/college education		34 (12.5)		
Nutritional status (BMI)		Frequency (%)	Description	
Less than 18.5		44 (16.2)	Underweight	
18.5-24.9		143 (52.8)	Normal weight	
25-29.9		57 (21.0)	Overweight	
30 or more		27 (10.0)	Obese	

The ages of participants ranged from 18 to 74 years (mean = 40.4 years), and there was no significant difference between the ART-naïve and ART-experienced cases. The majority of participants (123, 45.2%) were married. More than half of the participants had primary education, 5.5% of participants had no formal education, and only 12.5% of participants had university/college education.

Nutritional status was assessed through BMI. The mean BMI of the participants was 23 (SD 4.9), and there was no significant difference between the mean BMI of new cases (ART-naïve) compared to ART-experienced cases. Forty-four (16.2%) participants were underweight, and 27 (10.0%) were obese.

The mean CD4 cell count was 98 cells/µL (SD = 68 cells/µL), ranging from 7 to 200 cells/µL in the new cases group of participants. The mean viral load was 179,033 copies/mL (SD = 761,614 copies/mL), ranging from 1200 to 8,900,083 copies/mL.

**Figure 3:**
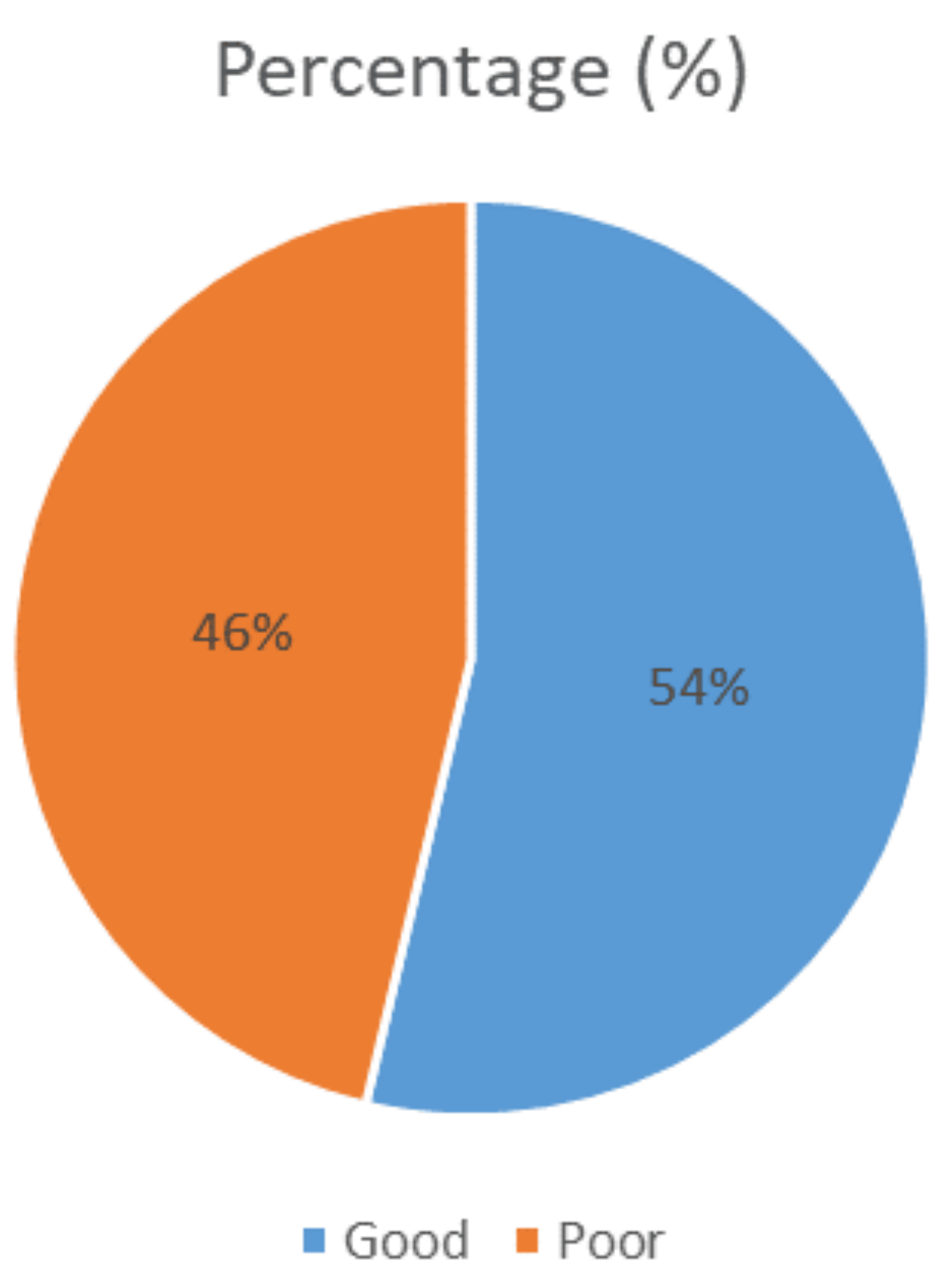
ART adherence among ART-experienced participants in the past six months. ART: antiretroviral therapy.

ART adherence among ART-experienced participants with viral load of ≥ 1000 copies/ml was poor in nearly half of the participants. This is illustrated in Figure 3.

Table 3 shows the ART regimen that was used by participants, of which most patients (two-thirds) were on first-line treatment at the time, and almost 33% were on second-line treatment of HIV.

**Table 3:** ART regimen used by participants during the period of the study. ART: antiretroviral therapy; ATV/r: atazanavir/ritonavir; AZT: zidovudine; 3TC: lamivudine; EFV: efavirenz; NVP: nevirapine; LPV/r: lopinavir/ritonavir; TDF: tenofovir disoproxil fumarate; FTC: emtricitabine.

Current ART regimen	Frequency	%	Cum. percent (%)
ATV/r-based combination	65	23.81	23.81
AZT+3TC+EFV	12	4.40	28.21
AZT+3TC+NVP	6	2.20	30.40
LPV/r-based combination	28	10.26	40.66
TDF+3TC+EFV	156	57.14	97.80
TDF+FTC+EFV	6	2.20	100.00
Total	273	100	100

Prevalence of asymptomatic cryptococcal antigenemia

Thirteen participants were found to be positive for CrAg, resulting in an overall CrAg prevalence of 4.8% (13/273; 95% CI: 2.6-8.0). Among the ART-naïve group, nine individuals tested CrAg positive, corresponding to a prevalence of 8.1% (9/111; 95% CI: 3.8-14.8). In the ART-experienced group, four participants were CrAg positive, yielding a prevalence of 2.5% (4/162; 95% CI: 0.7-6.2). A two-tailed Fisher’s exact test revealed a statistically significant difference in CrAg positivity between the ART-naïve and ART-experienced groups (p = 0.04).

Factors associated with asymptomatic cryptococcal antigenemia

Several factors did not show a statistically significant association with ACA; these included BMI, oral thrush, presence of skin lesions, productive cough, and poor adherence in the past six months. However, having a history of fever in the past two weeks had an odds ratio of 5.7 (1.8-17.9, p < 0.05), headache in the past two weeks had an odds ratio of 12.7 (2.7-58.4, p < 0.05), a history of weight loss in the past one month had an odds ratio of 8.1 (2.5-25.8, p < 0.05), a history of diarrhea in the past two weeks had an odds ratio of 8.4 (2.6-27.1, p < 0.05), and a history of hospital admission in the past 12 months had an odds ratio of 13.2 (4.0-43.1, p < 0.05), which was statistically associated with ACA. Table 4 illustrates the bivariate analysis of factors associated with positive serum CrAg.

**Table 4:** Bivariate analysis of factors associated with positive serum cryptococcal antigen (CrAg). ART: antiretroviral therapy; CrAg: cryptococcal antigen.

Variable	CrAg positive (%)	CrAg negative (%)	OR	95% CI	p-value
Symptoms	n (%)	n (%)			
Fever - YES	6 (46.2%)	34 (13.1%)	5.7	1.8 – 17.9	0.003
NO	7 (53.8%)	226 (86.9%)			
Headache - YES	3 (28.1%)	6 (2.3%)	12.7	2.7 – 58.4	0.006
NO	10 (76.9%)	254 (97.7%)			
Productive cough - YES	1 (7.7%)	40 (15.4%)	0.4	0.05 – 3.6	0.69
NO	12 (92.3%)	220 (84.8%)			
Weight loss - YES	8 (61.5%)	43 (16.5%)	8.1	2.5 – 25.8	0.0005
NO	5 (38.5%)	217 (83.5%)			
Diarrhea - YES	6 (46.2%)	24 (9.3%)	8.4	2.6 – 27.1	0.0002
NO	7 (53.8%)	236 (90.7%)			
BMI					
≤18	3 (23.1%)	41 (15.9%)	0.5	0.1 – 1.9	0.39
>18	10 (76.9%)	217 (84.1%)			
Signs					
Oral thrush - YES	2 (15.4%)	13 (5%)	3.5	0.6 – 17.2	0.15
NO	11 (84.6%)	247 (95%)			
Skin lesions - YES	3 (23.1%)	38 (15.1%)	0.6	0.2 – 2.2	0.43
CD4 count cells/µL					
0-100	8 (72.3%)	54 (48.7%)	0.4	0.1 – 1.4	0.21
101-200	3 (27.3%)	57 (52.3%)			
Recent viral load (copies/ml)					
1000-50000	2(33.4%)	109 (67.5%)	4.2	0.7 – 23.6	0.09
≥50000	4 (66.6%)	52 (32.3%)			
ART adherence in the past 6 months					
Poor	3 (75%)	72 (45.6%)	0.2	0.02 – 2.7	0.33
Good	1 (25%)	86 (54.4%)			
History of hospital admission in the past 12 months					
YES	8 (61.5%)	28 (10.8%)	13.2	4.0 – 43.1	0.00003
NO	5 (38.5%)	231 (89.2)			

Table 5 illustrates the logistic regression analysis conducted for factors that were significantly associated with ACA on univariate analysis. History of headache in the past two weeks and history of hospital admission in the past were found to statistically predict ACA.

**Table 5:** Multiple logistic regression analysis of factors associated with asymptomatic cryptococcal antigenemia.

Variable	OR	95% CI	p-value
Fever	2.7	0.6 – 11.8	0.16
History of hospital admission in the past 12 months	6.6	1.7 – 25.2	0.006
Weight loss	3.7	0.9 – 15.6	0.06
Headache	11.1	1.2 – 100.9	0.03
Diarrhea	3.9	0.9 – 16.5	0.05

Outcomes of asymptomatic cryptococcal antigenemia at 12 weeks

Overall, 45 participants were followed up for three months from the date of enrolment. For every CrAg-positive participant, two CrAg-negative participants were followed up through mobile telecommunication with them or their relatives. Six patients were added to account for loss of follow-up. All CrAg-positive patients completed three months of follow-up. Three outcomes were assessed: development of symptoms of meningitis (at least two symptoms of meningitis), hospital admission, and mortality.

Four (33.3%) participants out of 13 who were CrAg-positive at the beginning of the study developed symptoms of meningitis (Table 6). Four out of 13 patients in the CrAg-positive participants were admitted to the hospital, and there was one death. The likelihood of an adverse outcome was statistically higher (69%, p < 0.05) in participants who had a CrAg-positive test compared to those who had a negative CrAg test.

**Table 6:** Outcomes of asymptomatic cryptococcal antigenemia at 12 weeks of follow-up. CrAg: cryptococcal antigen.

Outcomes	CrAg status		P-value
	Positive (n = 13)	Negative (n = 30)	
Development of symptoms of meningitis (%)	4 (33.3%)	1 (3.3%)	-
Hospital admission (%)	4 (33.3%)	1 (3.3%)	-
Deaths (%)	1 (8.3%)	0	-
Loss of follow-up (%)	1 (7.7%)	3 (9.4%)	
Likelihood of adverse outcomes (%)	9 (69%)	2 (4.7%)	0.00001

## Discussion

This is the first study in Tanzania to estimate the prevalence of ACA in both ART-naïve and ART-experienced patients in three HIV clinics in Dar es Salaam city. In this study, the overall prevalence of ACA among ART-naïve and ART-experienced participants was 4.8%. This prevalence was lower in comparison to the two previous studies in Ethiopia by Beyene et al. and Alemu et al., which reported prevalence rates of 10.2% and 8.4%, respectively [19,21]. This difference might have been due to geographical variations in cryptococcal antigenemia and the non-exclusion of patients with symptoms of meningitis.

Viral load of more than 1000 copies/ml was used as a criterion to include ART-experienced cases, where CrAg prevalence in this group was 2.5% statistically less than ART-naïve cases, in which the prevalence was 8.1% (p = 0.04). This difference was similar to the findings by Beyene et al., where the majority of CrAg-positive patients were ART-naïve [21]. However, another study did not find any significant difference in proportion between ART-naïve and ART-experienced patients [20].

The prevalence of 8.1% in ART-naïve patients is similar to other large studies in South Africa [28], where the prevalence of cryptococcal antigenemia was 7%, and Indonesia [15], where the prevalence was 7.1%. In comparison to a study conducted in the two countries of Tanzania and Zambia, whereby Dar es Salaam was one of the cities where the study was conducted, the proportion of cryptococcal antigenemia among ART-naïve patients was 5% [23]. However, the latex agglutination method was used to screen for cryptococcal antigenemia [23], in comparison to the LFA method, which was used in our study. The sensitivity and specificity of the CrAg-LFA test are higher compared to the latex agglutination method. It is simple to use with a short turnaround time and does not require preheating of the sample [16].

The present results have demonstrated that there are ACA cases in ART-experienced patients; however, it may not be cost-effective to screen everyone with viral loads > 1000 copies/ml. In addition, the WHO had justified screening when the prevalence of CrAg was >3%, hence demonstrating the need for a more selective, risk-driven, and individualized screening strategy in ART-experienced patients with a high index of suspicion.

The index study also revealed that the history of hospital admission in the past 12 months was associated with cryptococcal antigenemia. This finding was similar to a study by Hailu et al. [20]. A history of hospitalization might signify clinical deterioration, which may result from treatment failure, contributing to the emergence of opportunistic infections such as *Cryptococcus*.

There were some symptoms that were associated with cryptococcal antigenemia in univariate analysis, but only a history of recent headache in the past week showed a significant association with cryptococcal antigenemia. Headache is one of the symptoms of meningitis, but it is less specific on its own. Notably, one study conducted in Uganda had revealed that possessing at least two or more symptoms/signs had higher specificity than a single symptom [12].

The index study found that low BMI was not associated with ACA, unlike two other studies [12]. One of which showed a significant association between CrAg antigenemia and having a BMI of less than 18 kg/m^2^ [12], whereas another study [16] found that cryptococcal antigenemia was associated with having a BMI of less than 15.4 kg/m^2^. Our study was done in an urban setting compared to a previous study [12], which was done in rural Uganda. The index study showed no association between low BMI and ACA. One possible explanation might be the better nutrition status of urban populations compared to rural populations, which may have influenced the BMI in our study participants who were from an urban setting [29]. More than half of our patients had used ART for more than six months, which, in addition, may have improved the nutritional status, compared to participants in previous studies that were conducted in ART-naïve patients only.

In our study, the proportion of CrAg-positive patients among the ART-naïve participants was not different when comparing CD4 count between 0-100 and 101-200 cells/µL. The finding is similar to two studies; the first conducted in Namibia [13], and the other in northern Tanzania. This indicates that there may be a need to consider individualized cutoff CD4 cell counts for CrAg screening among ART-naïve patients. However, these findings should be interpreted with caution due to a limited sample size and a relatively small number of ART-naïve participants in our study. Nevertheless, other larger studies had demonstrated that CrAg positivity is more prevalent among individuals with CD4 counts below 100 cells/µL [12,30,31].

In the present study, there was one death at three months; however, the probability of an adverse event (development of meningitis, hospitalization, and death) was statistically higher in CrAg-positive patients than in CrAg-negative patients. Prior studies that analyzed short-term outcomes of cryptococcal antigenemia in ART-naïve patients were both retrospective; one of which was conducted in northern Tanzania, in which there was no difference in mortality between CrAg-negative and CrAg-positive patients at six months [7]. While the one in Uganda showed 23% mortality at three months [12].

Although participants in our study with positive cryptococcal antigenemia were started on treatment, there was still a poor outcome at the 12th week. This could be due to several reasons, including adequacy and efficacy of treatment, severity of infection, or concomitant other infections.

It was also observed that the ART adherence among ART-experienced patients with viral load of >1000 copies/mL was very poor, which is usually evaluated in every clinic visit and reported on clinic documents. In ART-experienced patients with a viral load of 1000 copies/mL, 43.6% were observed to have poor adherence. These findings were similar to another study done in northern Tanzania, where the non-adherence was 42% among ART users [32]. This predisposed this subgroup to drug resistance and treatment failure that could potentially result in clinical deterioration and the emergence of opportunistic infections such as *Cryptococcus* infection.

The screening test that we used was simple to use, and the participants got their results immediately after the interview. The cost of the screening test was relatively inexpensive; it costs about 1 US$ per test and was reported to have high sensitivity and specificity in previous studies. It is, therefore, a practical tool for use in clinical settings and can be easily integrated into the routine care of HIV patients in CTCs.

Adoption of a risk-based strategy alongside CD4 count for screening of ART-naive HIV patients for ACA should be considered. Since a considerable subgroup of ART-experienced patients had ACA and were also at risk of poor outcomes, a more robust risk-based assessment and screening guideline in this cohort is warranted. In addition, a simple algorithm can be utilized to screen cryptococcal antigenemia in ART-experienced patients in ambulatory care. The algorithm should take into account the index of suspicion of cryptococcal infection depending on several predetermined factors.

Limitations

This study had several limitations. One was the reliance on recent laboratory results for CD4 counts and viral loads rather than performing fresh tests at the time of screening. However, this approach reflects actual practice in most HIV clinics in Tanzania, where obtaining these results can take time.

A follow-up at three months was conducted via mobile phones, which may have affected data quality due to the lack of face-to-face contact with participants. Nevertheless, most study participants and their next of kin were cooperative and forthcoming in sharing information over the phone.

This study was not sufficiently powered to determine differences in outcomes between ART-naïve and ART-experienced patients with cryptococcal antigenemia at 12 weeks; therefore, further research in this area is recommended.

Additionally, the study was conducted in only three HIV clinics, which may limit the generalizability of the findings to the entire country. Larger studies are recommended, particularly to evaluate the utility of screening for cryptococcal infection in ART-experienced patients with virological failure.

## Conclusions

The prevalence of ACA in both ART-naïve patients with CD4 counts of ≤ 200 cells/µL and ART-experienced patients with viral load ≥1000 copies/mL was 4.8% in the three HIV clinics in Dar es Salaam. The prevalence of ACA in ART-experienced cases for more than six months was lower compared to new cases of HIV with a CD4 count less than 200 cells/µL. There is evidence of a potential benefit of individualized screening for ACA in patients with a history of hospitalization in the past 12 months and clinical deterioration, besides CD4 counts alone. Having a CrAg positivity was a predictor of an adverse outcome at three months. Screening of a subgroup of ART-experienced patients with risk factors may reduce mortality associated with cryptococcal meningitis in ART-experienced patients.
